# Combination therapy success in refractory pediatric visceral leishmaniasis with hemophagocytic lymphohistiocytosis: A case report

**DOI:** 10.1097/MD.0000000000047374

**Published:** 2026-01-30

**Authors:** Rahmah Zafar Masood, FNU Nikeeta, Aqsa Altaf, George Jabrieh

**Affiliations:** a CMH Lahore Medical College, Lahore, Pakistan; b Ghulam Muhammad Mahar Medical College, Sukkur, Pakistan; c Shaheed Mahutarma Benazir Bhutto University, Larkana, Pakistan; d Jinnah Sindh Medical University, Karachi, Pakistan; e Palestine Polytechnic University, College of Medicine and Health Sciences, Hebron, Palestine.

**Keywords:** hemophagocytic lymphohistiocytosis, multidrug therapy, pediatric leishmaniasis, treatment resistance, visceral leishmaniasis

## Abstract

**Rationale:**

Visceral leishmaniasis (VL) is a parasitic disease with high mortality if untreated. Its association with secondary hemophagocytic lymphohistiocytosis (HLH) is rare but life-threatening, posing diagnostic and therapeutic challenges.

**Patient concerns:**

An 11-month-old female presented with fever, abdominal distension, and pancytopenia.

**Diagnoses:**

Bone marrow examination confirmed VL, and laboratory findings indicated secondary HLH.

**Interventions:**

Initial treatment with sodium stibogluconate and liposomal amphotericin B was unsuccessful. Following a multidisciplinary review, miltefosine was initiated alongside repeat doses of liposomal amphotericin B.

**Outcomes:**

The combination therapy resulted in rapid clinical and hematologic improvement.

**Lessons:**

This case highlights the importance of early recognition of HLH in VL, the role of multidisciplinary management, and the potential efficacy of combination therapy in refractory pediatric cases, particularly in resource-limited settings.

## 1. Introduction

Trypanosomatid protozoan parasites belonging to the *Leishmania donovani* complex are the cause of visceral leishmaniasis (VL). If left undiagnosed and untreated, the illness has a >90% case fatality rate in humans within 2 years.^[1]^ Although it is endemic in more than 60 countries, it remains a serious public health concern. However, studies have linked more than 90% of cases to just 7 nations: Brazil, Ethiopia, India, Kenya, Somalia, South Sudan, and Sudan.^[2]^ Disseminated *Leishmania* parasite infection throughout the reticuloendothelial system is the cause of VL. Pancytopenia, hepatomegaly, hypergammaglobulinemia, splenomegaly, prolonged irregular fever, and weight loss are typical clinical characteristics.^[3]^ In rare situations, VL can be exacerbated by hemophagocytic lymphohistiocytosis (HLH), a hyperinflammatory condition characterized by excessive immunological activation that, if left untreated, results in severe cytopenias, organomegaly, and high mortality. Although some people with HLH are genetically predisposed to the illness, the majority of instances are random. In either situation, infection or impaired immune hemostasis may cause an episode of HLH.^[4]^ HLH in the setting of VL poses significant diagnostic and therapeutic issues due to overlapping clinical characteristics and the necessity for immediate, intense care.^[5]^ This case report highlights the difficulties in detecting and treating a pediatric child with VL exacerbated by secondary HLH. The case highlights the importance of a multidisciplinary approach in managing such complex illnesses. It draws attention to the therapeutic challenges that arise during treatment, such as treatment resistance to 1st-line medications and the eventual success of using alternative therapies.

## 2. Case presentation

An 11-month-old female from Pakistan presented with a 2-week history of lethargy, high-grade fever (103°F), and abdominal distension. Born at term via elective lower segment cesarean section with no significant perinatal history, she was exclusively breastfed until 6 months. She had received all vaccinations per the Expanded Program on Immunization. Physical examination revealed a febrile, moderately anemic child with gross abdominal distension, a palpable spleen 7 cm below the left costal margin, and a palpable liver 6 cm below the right costal margin. Microscopic examination of the bone marrow aspirate revealed hypercellular marrow with prominent histiocytic hyperplasia. Numerous histiocytes were seen engorged with intracellular amastigote forms of *Leishmania* spp., recognized by their round shape, size of 2 to 4 μm, and the presence of a characteristic rod-shaped kinetoplast adjacent to the nucleus. Extracellular amastigotes were also noted, confirming the diagnosis of VL. Based on clinical and laboratory findings, the patient was also diagnosed with secondary HLH. Other possible causes of prolonged fever, hepatosplenomegaly, and pancytopenia, including malaria, typhoid fever, and hematologic malignancies, were considered and excluded through negative peripheral smear for malarial parasites, sterile blood cultures, and absence of blast cells on bone marrow examination. Despite initial treatment with sodium stibogluconate and liposomal amphotericin B, the patient’s condition deteriorated, prompting the addition of miltefosine to the treatment regimen, which ultimately led to clinical improvement. This case highlights the challenges in diagnosing and managing VL with secondary HLH in a pediatric patient. Serial laboratory investigations demonstrated persistent pancytopenia with progressively worsening thrombocytopenia and anemia, along with elevated ferritin and triglyceride levels at the time of HLH diagnosis. These findings are summarized in Table 1.

**Table 1:** Key laboratory findings at different stages of hospitalization.

Parameter	Initial presentation	Post 1st-line failure	Post 2nd-line failure (HLH diagnosis)	At follow-up/discharge
Hemoglobin (g/dL)	6.0	6.0	6.0	10.5
Platelets (/µL)	28,000	–	17,500	150,000
Reticulocyte (%)	0.4	–	0.6	2.0
Ferritin (ng/mL)	–	–	2759	87
Triglycerides (mg/dL)	–	–	845	149
Fibrinogen (mg/dL)	–	–	350	210

Follow-up laboratory evaluation after completion of combination therapy demonstrated progressive hematologic recovery. Hemoglobin and platelet counts showed steady improvement over the following weeks, with complete normalization within 1 month of therapy. Ferritin levels, which were markedly elevated at the time of HLH diagnosis, declined rapidly after initiation of miltefosine and repeat liposomal amphotericin B, reaching the normal range during outpatient follow-up. These trends reflected both adequate control of the VL infection and resolution of secondary HLH.

The patient’s clinical course, including interventions and outcomes at each stage, is outlined in Table 2.

**Table 2:** Timeline of management and clinical outcomes.

Time point	Clinical status/findings	Intervention	Outcome
Day 0	Fever, hepatosplenomegaly, pancytopenia; bone marrow + for *Leishmania*	Sodium stibogluconate (8 days) + supportive transfusions	No improvement; persistent symptoms
2 wk later	Persistent fever, pancytopenia, hepatosplenomegaly	Liposomal amphotericin B	No significant response
1 mo later	Persistent fever, pancytopenia, hepatosplenomegaly; HLH diagnosed (Ferritin 2759 ng/mL, TG 845 mg/dL, Fibrinogen 350 mg/dL)	Multidisciplinary review; plan for combination therapy	–
After 3 doses	Afebrile, improved blood counts, and spleen size decreasing	Miltefosine + liposomal amphotericin B	Marked clinical improvement
Follow-up	Liver palpable 2 cm, spleen 2 cm; active and alert	Continued follow-up	Full recovery with normalization of laboratory parameters (see Table 1)

## 3. Discussion

VL is an issue of concern in endemic areas, particularly among children from poor socioeconomic backgrounds in South Asia and East Africa.^[6]^ The development of secondary HLH added to the case’s complexity. This uncommon yet life-threatening complication can arise with a variety of diseases, including *leishmania*. HLH is caused by an unregulated immune response to various stimuli, such as pathogenic organisms, tissue injury, metabolic products, and others, which is based on an underlying genetic or acquired incapacity to cope appropriately with the trigger.^[7]^ Activated lymphocytes and macrophages release large amounts of pro- and anti-inflammatory cytokines, chemokines, and other chemicals, resulting in significant clinical and laboratory results.^[7]^

VL itself induces a potent cytokine-driven inflammatory response, with marked elevations in interferon-γ, tumor necrosis factor-α, interleukin-6, and interleukin-10. Persistent antigenic stimulation by *Leishmania* amastigotes leads to continuous activation of macrophages and T lymphocytes, overwhelming normal immune regulatory mechanisms. This cytokine storm not only contributes to pancytopenia and organomegaly but also directly drives the uncontrolled macrophage activation and hemophagocytosis that characterize secondary HLH. Thus, VL provides a strong and sustained immunologic trigger capable of precipitating the full hyperinflammatory cascade of HLH.

The clinical features of VL overlap with those of HLH; therefore, identifying leishmaniasis as a trigger for the condition can be difficult, especially in endemic locations. As a result, a delay in diagnosing HLH linked with VL, most likely due to clinical similarities, can result in severe consequences and mortality in up to 90% of patients without HLH-specific therapy.^[8]^

In this case, the patient originally did not react to 1st-line treatment with sodium stibogluconate and liposomal amphotericin B, which are typical therapies for VL.^[9]^ Treatment resistance in VL, especially when accompanied by HLH, emphasizes the significance of exploring other treatment options. The subsequent use of miltefosine, an oral antileishmanial drug with a broad range of action, in conjunction with repeated doses of liposomal amphotericin B, resulted in clinical improvement, demonstrating the efficacy of combination treatment in resistant patients.^[10]^

The identification of secondary HLH complicated the situation since it required not only controlling the Leishmania infection but also managing a hyperinflammatory condition. Early detection of HLH and the timely beginning of treatment to address both diseases are crucial to improving outcomes in such instances.^[11]^ In this case, a multidisciplinary team, comprising infectious disease experts and hematologists, was critical in directing the management strategy, which ultimately resulted in a positive outcome. Supportive care, including transfusions and close monitoring for treatment-related adverse effects, was critical to the patient’s recovery. The ultimate success of miltefosine in combination with liposomal amphotericin B emphasizes the necessity of personalized treatment strategies in pediatric VL patients, particularly those who develop secondary problems, such as HLH.^[12]^ This instance highlights the need for more research to enhance treatment techniques in controlling VL complicated by HLH, particularly in resource-constrained settings. Early detection of treatment failure and a flexible, multidisciplinary treatment strategy are critical for addressing difficult situations like this 1 and, ultimately, improving patient outcomes.

## 4. Limitations

A limitation of this report is the absence of photomicrographs of the diagnostic bone marrow smear or imaging studies, which would have provided further visual documentation. Despite this, the definitive laboratory and clinical findings robustly support the diagnoses of VL and secondary HLH.

## 5. Conclusion

This case demonstrates the challenges of treating VL when it is accompanied by secondary HLH. The initial failure of 1st-line medicines emphasizes the difficulties in managing treatment-resistant VL, especially in pediatric patients. The ultimate effectiveness of miltefosine and extra dosages of liposomal amphotericin B highlights the need for an individualized therapy approach in such patients. Early identification of secondary HLH, along with an integrated strategy, was critical in guiding the patient’s recovery. This example highlights the need for more study into optimum treatment procedures for children with VL complicated by HLH, particularly in resource-constrained areas where access to sophisticated medicines may be limited.

## Author contributions

**Conceptualization:** Rahmah Zafar Masood.

**Methodology:** Rahmah Zafar Masood, FNU Nikeeta.

**Writing – original draft:** Rahmah Zafar Masood, FNU Nikeeta, Aqsa Altaf.

**Data curation:** FNU Nikeeta.

**Writing – review & editing:** Aqsa Altaf, George Jabrieh.
